# Central corneal sensitivity after small incision lenticule extraction versus femtosecond laser-assisted LASIK for myopia: a meta-analysis of comparative studies

**DOI:** 10.1186/s12886-015-0129-5

**Published:** 2015-10-24

**Authors:** Miao He, Wenyong Huang, Xingwu Zhong

**Affiliations:** Zhongshan Ophthalmic Center, State Key Laboratory of Ophthalmology, Sun Yat-Sen University, 54SXianlie Road, Guangzhou, 510060 China; Hainan Eye Hospital, Zhongshan Ophthalmic Center, Sun Yat-sen University, Haikou, 570311 China

**Keywords:** Corneal sensitivity, Small incision lenticule extraction, Femtosecond laser-assisted laser in situ keratomileusis, Myopia, Meta-analysis

## Abstract

**Background:**

The aim of this study was to evaluate central corneal sensitivity after small incision lenticule extraction (SMILE) versus femtosecond laser-assisted laser in situ keratomileusis (FS-LASIK) for myopia.

**Methods:**

Eligible studies were identified by systematically searching PubMed, the Cochrane Library, Embase and the Chinese Biomedicine Database. Central corneal sensitivity after SMILE versus FS-LASIK was assessed by the pooled weighted mean differences (WMDs) of the reduction from pre-operation levels to 1 week, 1, 3 and 6 months following the operation. The quality of the included literature was assessed by the Quality Index. Data were synthesized by Stata 12.0 SE for Windows.

**Results:**

Five studies involving 245 patients (363 eyes; 189 eyes in the FS-LASIK group and 174 eyes in the SMILE group) were included in the meta-analysis. The pooled results showed that there were no significant differences in central corneal sensitivity between FS-LASIK and SMILE before surgery (WMD = 0 mm, 95 % CI: −0.23 to −0.23, *p* = 0.998). At 1 week, 1 and 3 months after surgery, the central corneal sensitivity after FS-LASIK was lower than with SMILE (WMD = −17.35 mm, 95 % CI: −26.54 to −8.16, *p* <0.001; WMD = −17.52 mm, 95 % CI: −25.10 to −9.94, *p* <0.001; WMD = −14.64 mm, 95 % CI: −20.08 to −9.21, *p* <0.001, respectively). However, central corneal sensitivity after FS-LASIK was similar with SMILE 6 months after surgery (WMD = −2.02 mm, 95 % CI: −4.23 to 0.19, *p* = 0.074).

**Conclusion:**

Central corneal sensitivity exhibited a small decrease and a faster recovery after the SMILE procedure compared to FS-LASIK during the first three postoperative months. Corneal sensitivity after SMILE and FS-LASIK was similar at 6 months after surgery. However, these results should be interpreted with caution due to the limited number of studies.

## Background

The femtosecond laser (FS) is characterized by ultrafast (10^−15^ s) pulses, smaller shock waves, highly precise tissue cleavage and a reduced zone of collateral damage [1]. Since it was introduced to the market in 2002, its use has steadily burgeoned [2]. Currently, corneal flap creation in laser in situ keratomileusis (LASIK) surgery is mostly performed using the FS laser. A reduction of corneal sensation and dry eye are the most common complications after all types of corneal refractive surgery. The reasons for these postoperative conditions include damage to the corneal nerves and goblet cells at the limbus, reduced blinking reflex and tear production and increased tear evaporation [3]. Because FS-LASIK generates more consistent and predictable flap diameters and thicknesses than microkeratome, the incidence of dry eye decreased and the recovery of corneal sensation after FS-LASIK was faster than after traditional LASIK [4].

Small incision lenticule extraction (SMILE) is a new corneal refractive surgery for myopia using FS [5]. It is an all-in-one process in which the flap is replaced by a cap and an intrastromal lenticule is generated between two photodisruption planes. The lenticule will be removed from a 2.0 to 4.0 mm arcuate side cut which is shorter than that of a LASIK flap. Theoretically, SMILE is a kind of “flapless” corneal refractive surgery and is associated with less flap-related complications. SMILE damages fewer corneal nerves and preserves more original corneal biomechanics in comparison with FS-LASIK and traditional LASIK. Recent studies have compared corneal sensitivity after SMILE and after FS-LASIK [6–10]. However, the results were controversial and the studies were less convincing because of the small sample sizes and other research design limitations. Therefore, a meta-analysis is imperative for summarizing results from different studies [11]. The aim of this study was to perform a systematic review and meta-analysis to evaluate the changes of corneal sensation after SMILE and FS-LASIK.

## Methods

This was a meta-analysis, thus, the requirements for an ethics statement and consent forms were not needed. This study was conceived, conducted and reported according to the Preferred Reporting Items for Systematic Reviews and Meta-Analyses (PRISMA) statement [12].

### Literature search strategy

Prospective comparative studies were identified through a systematic search of PubMed, the Cochrane Library, EMBASE and the Chinese Biomedicine Database (All searches were conducted prior to March 2015). The search terms included: myopia, near-sighted, short-sighted, small incision lenticule extraction, smile and SMILE. Websites of professional associations and the Google Scholar search engine were also searched. Language restrictions were not used. The references of reviews and the included studies were also screened for additional studies that were not included in the computerized databases. Two reviewers determined the trial eligibility independently. First, the titles and abstracts of the obtained publications were screened. Then, the full articles of the remaining identified publications were scrutinized. Only trials meeting the inclusion criteria were assessed for methodological quality.

### Inclusion criteria and outcomes

The following inclusion criteria were used in the present meta-analysis: (1) study design: randomized or non-randomized clinical trials; (2) population: patients with myopia (range from −2 to -10D); (3) intervention: SMILE versus FS-LASIK; and (4) outcome variables: corneal sensitivity or corneal sensation. The outcomes were measured by the reductions of central corneal sensitivity from preoperative levels to levels at 1 week, 1, 3 and 6 months after surgery. Meeting abstracts with insufficient data, duplicate publications, letters and reviews were excluded.

### Data extraction

Data extraction was performed independently by two reviewers using a customized form. Any disagreement was resolved by discussion. The following information was extracted: the name of the first author, the publication year, the study design, interventions, the trial location, the follow-up durations, the number of subjects, the patients’ ages, preoperative central corneal sensitivity and preoperative spherical equivalent. If there were multiple reports for a particular study, data from the most recent publication were extracted.

### Assessment of methodology quality

The methodological quality of each study was assessed using the Quality Index, which can be used for the assessment of qualities of randomized and non-randomized clinical trials [13]. The Quality Index is composed of five main sections (Reporting, External Validity, Bias, Confounding and Power). Each section has several assessment standards. The full score of the Quality Index is 32 points. Two authors subjectively reviewed all studies and assigned a value of “yes,” “no,” or “unclear” for each section and finally calculated the total points of each included trial. Studies with a quality score of ≥16 were considered to have adequate quality [14].

### Statistical analysis

The outcome measures were assessed on an intention-to-treat (ITT) basis, the ITT population was comprised of all patients who received refractive surgery and provided a valid baseline measurement. Considering not all clinical characteristics were similar between groups, it was assumed that heterogeneity was present even when no statistical significance was identified; thus, we decided to combine data with a random effects mode [15]. For continuous outcomes, the weighted mean difference (WMD) was calculated. All outcomes were reported with *P* values and 95 % confidence intervals (CIs). Statistical heterogeneity among studies was assessed with the χ^2^ and I^2^ tests. An I^2^ value greater than 50 % indicates significant heterogeneity [16]. The overall effect was determined to be statistically significant with *P* <0.05. The analysis was conducted using the StataSE software package (Version 12.0; Stata Corp., College Station, TX).

### Sensitivity analysis and publication bias

To evaluate the robustness of the results, each study in the meta-analysis was excluded in turn to expose the influence of the individual studies on the pooled estimates, which was called leave-one-out analysis [14]. Potential publication bias was assessed visually with a funnel plot and statistically with the Egger’s and Begg’s tests [17, 18].

## Results

### Literature search

**Fig. 1:**
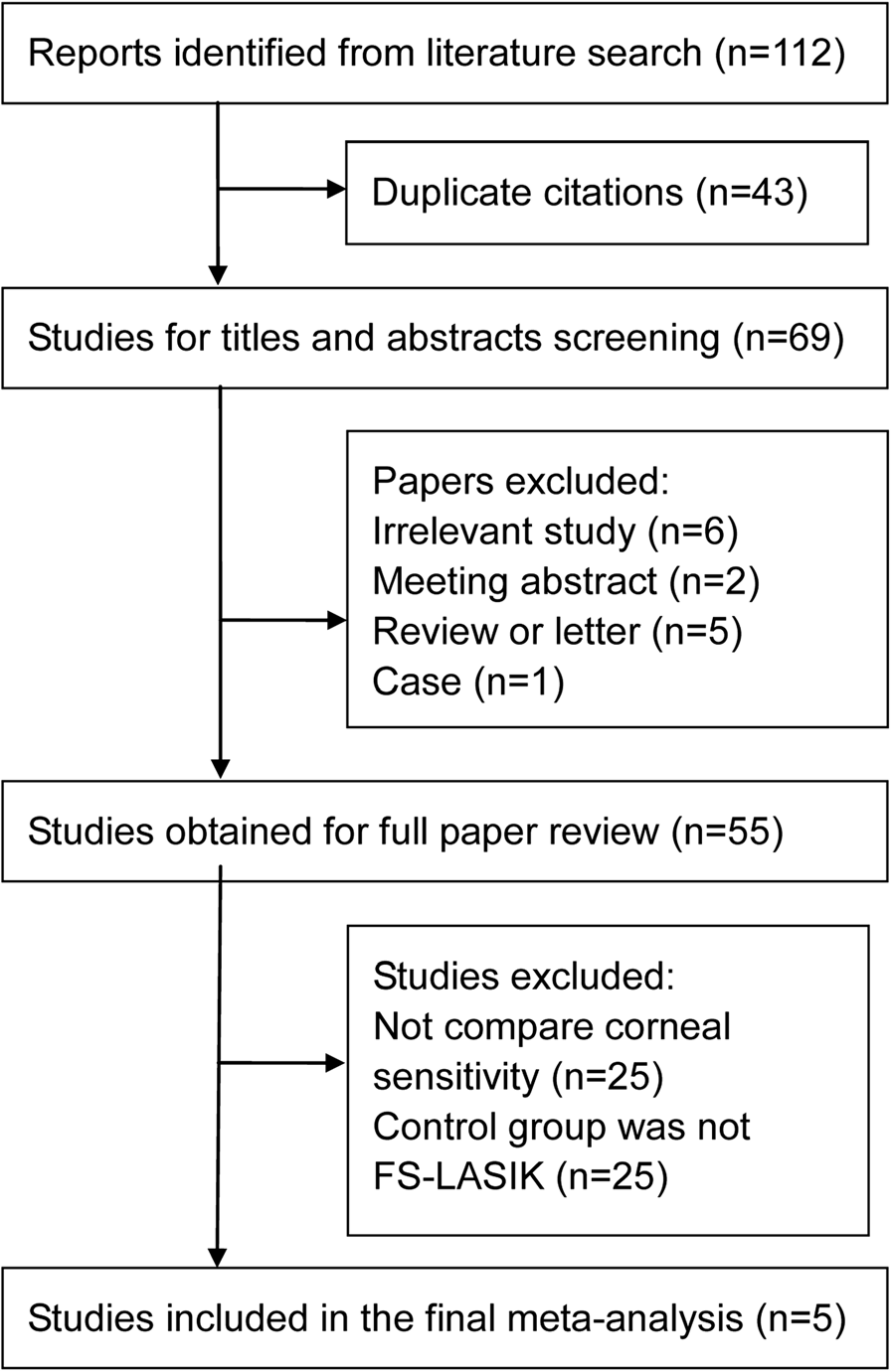
Flow diagram of the study selection

Figure 1 shows the detailed steps of the study selection process. Initially, 112 potentially eligible studies were retrieved from the electronic databases. After excluding 43 duplicate reports, 69 papers underwent title and abstract screening. Fourteen articles were excluded due to two being abstracts, four were reviews, one was a case, one a letter and there were six with irrelevant topics. After reviewing the full texts, 25 papers were further excluded because they compared SMILE with other surgeries, and 25 studies did not report corneal sensitivity. Finally, five studies [6–10] that met our inclusion criteria were included in the meta-analysis.

### Characteristics of eligible studies

Table 1 summarizes the main characteristics of all eligible studies. The studies were published in 2013 or 2014. A total of 245 patients (363 eyes) were evaluated, with 189 eyes in the FS-LASIK group and 174 eyes in the SMILE group. The mean age from the five different studies ranged from 25.44 to 28.3 years. Of the five prospective comparative trials, four were non-randomized studies and one was a randomized design. The durations of follow-ups were 6 months in three studies and 3 months in two studies. Additionally, four studies were conducted in China and one study in Turkey.

**Table 1:** Characteristics of prospective comparative studies included in the meta-analysis

First author	Year	Location	Design	Follow-up	Intervention	No. of eyes	Mean age(y)	Preoperative central corneal sensitivity	Preoperative SE(D)
Li(a)	2014	China	Non-randomized	6 m	FS-LASIK	33	27.33 ± 6.58	57.27 ± 6.26	NA
					SMILE	38	28.21 ± 7.04	58.16 ± 3.37	NA
Wei	2013	China	Non-randomized	3 m	FS-LASIK	54	25.44 ± 7.15	58.1 ± 4.3	NA
					SMILE	61	27.44 ± 6.52	56.6 ± 4.5	NA
Demirok	2013	Turkey	Randomized	6 m	FS-LASIK	28	26.2 ± 4.4	56.2 ± 5.0	4.00 ± 1.40
					SMILE	28	26.2 ± 4.4	56.8 ± 4.7	3.90 ± 1.50
Gao	2014	China	Non-randomized	3 m	FS-LASIK	32	22.75 ± 4.28	57.66 ± 3.25	NA
					SMILE	15	24.53 ± 4.05	58.48 ± 2.84	NA
Li(b)	2013	China	Non-randomized	6 m	FS-LASIK	42	28.3 ± 5.5	58.06 ± 3.8	8.46 ± 2.15
					SMILE	32	27.1 ± 4.0	58.26 ± 4.5	6.56 ± 1.28

The results were performed as Mean ± SD

*FS-LASIK* femtosecond laser-assisted laser in situ keratomileusis, *SMILE* small incision lenticule extraction, *y* year, *SE* spherical equivalent, *D* diopter, *m* month, *NA* not available

### Quality assessment

Table 2 summarizes the quality assessment. In the Reporting section, all of these trials received scores of more than nine points, which meant that they all clearly described the details of the trials. For the External Validity section, all studies received full scores, which meant they had a good representativeness of the researchers, subjects and devices. Concerning the Bias section, none of the studies provided information on the procedures for allocation concealment. Furthermore, the blinding of patients, personnel and assessors were not reported, therefore, the scores were relatively low. With respect to the Confounding section, all were given low scores except one study [6], which was the only randomized trial, the other studies failed to use randomization. In the Power section, most studies had great power since the statistical method was appropriate and the main outcomes were accurately calculated. For the Quality Index score, all studies were over 16 points, indicating adequate quality.

**Table 2:** Evaluation of the quality of the included studies in this meta-analysis using Quality Index

First author	Year	Quality score component					Score
		I	II	III	IV	V	Over all
Li(a)	2014	10	3	4	3	5	25
Wei	2013	9	3	4	2	5	23
Demirok	2013	9	3	4	4	5	25
Gao	2014	9	3	4	2	5	23
Li(b)	2013	10	3	4	3	5	25

I: Reporting; II: External Validity; III: Bias; IV: Confounding; V: Power

### Efficacy analysis

Table 3 shows the meta-analysis of the reduction of central corneal sensitivity between FS-LASIK and SMILE. Before refractive surgery, there was no difference in central corneal sensitivity between the two surgery groups (*p* = 0.998, 95 % CI: −0.23 to 0.23). Postoperatively, at 1 week, 1 and 3 months, the central corneal sensitivity in the SMILE group was higher than that of the FS-LASIK group. However, at the 6th postoperative month, the difference in the central corneal sensitivity between the two groups was not statistically significant. No significant heterogeneity was observed before surgery between the two groups (*p* = 0.310, I^2^ = 16.30 %). However, there was significant heterogeneity between the two surgery groups postoperatively at 1 week (*p* <0.001, I^2^ = 93.20 %), 1 month (*p* <0.001, I^2^ = 90.40 %), 3 months (*p* <0.001, I^2^ = 82.90 %), and 6 months (*p* = 0.013, I^2^ = 68.60 %).

**Table 3:** The results of the meta-analysis for central corneal sensitivity after FS-LASIK and SMILE

Time points	WMD (95 % CI)			Test for heterogeneity			Test for overall effect	
	Estimate	Lower	Up	*χ* ^2^	I^2^	*P*	Z	*P*
Pre-operation	0.00	−0.23	0.23	4.78	16.30 %	0.310	0.00	0.998
One week after surgery	−17.35	−26.54	−8.16	59.08	93.20 %	<0.001	3.70	<0.001
One month after surgery	−17.52	−25.10	−9.94	41.67	90.40 %	<0.001	4.53	<0.001
Three months after surgery	−14.64	−20.08	−9.21	23.35	82.90 %	<0.001	5.28	<0.001
Six months after surgery	−2.02	−4.23	0.19	12.72	68.60 %	0.013	1.79	0.074

*FS-LASIK* femtosecond laser-assisted laser in situ keratomileusis, *SMILE* small incision lenticule extraction, *WMD* Weighted mean differences which were computed by using a random effects model

**Table 4:** Results of leave-one-out analysis in central corneal sensitivity after FS-LASIK and SMILE

Study excluded	WMD (95 %)			Test for heterogeneity			Test for overall effect	
	Estimate	Lower	Up	*χ* ^2^	I^2^	*P*	Z	*P*
One week after surgery								
Li(a)	−19.56	−29.72	−9.40	48.21	93.80 %	<0.001	3.77	<0.001
Wei	−14.85	−25.08	−4.63	37.04	91.90 %	<0.001	2.85	<0.001
Demirok	−17.97	−29.26	−6.69	56.87	94.70 %	<0.001	3.12	0.002
Gao	−14.47	−24.74	−4.20	44.12	93.20 %	<0.001	2.76	0.006
Li(b)	−19.90	−28.96	−10.84	32.36	90.70 %	<0.001	4.13	<0.001
One month after surgery								
Li(a)	−19.85	−27.61	−12.09	28.97	89.60 %	<0.001	5.01	<0.001
Wei	−15.24	−23.63	−6.86	28.78	89.60 %	<0.001	3.56	<0.001
Demirok	−18.49	−27.62	−9.36	37.52	92.00 %	<0.001	3.97	<0.001
Gao	−15.15	−23.34	−6.96	27.49	89.10 %	<0.001	3.63	<0.001
Li(b)	−18.81	−27.702	−9.91	33.19	91.90 %	<0.001	4.14	<0.001
Three months after surgery								
Li(a)	−15.77	−21.61	−9.94	18.34	83.60 %	<0.001	5.30	<0.001
Wei	−12.80	−19.34	−6.25	16.45	81.80 %	0.001	3.83	<0.001
Demirok	−15.45	−21.60	−9.30	18.89	84.10 %	<0.001	4.92	<0.001
Gao	−12.85	−19.65	−6.06	18.34	83.60 %	<0.001	3.71	<0.001
Li(b)	−16.16	−21.61	−10.71	15.45	80.60 %	0.001	5.81	<0.001
Six months after surgery								
Li(a)	−14.64	−20.08	−9.21	23.35	82.90 %	<0.001	5.28	<0.001
Wei	−2.75	−6.20	0.69	12.34	75.70 %	0.006	1.57	0.117
Demirok	−2.45	−5.40	0.51	12.56	76.10 %	0.006	1.62	0.105
Gao	−2.93	−5.29	−0.58	6.26	52.10 %	0.100	2.44	0.015
Li(b)	−1.29	−3.29	0.72	8.26	63.70 %	0.041	1.26	0.208

*WMD* Weighted mean differences which were computed by using a random effects model, *FS-LASIK* femtosecond laser-assisted laser in situ keratomileusis, *SMILE* small incision lenticule extraction

### Sensitivity analysis and publication bias

The results of the leave-one-out analysis showed that these exclusions did not alter the results of previous analyses at 1 week, 1 and 3 months after surgery (Table 4). At 6 months postoperatively, the combined results were changed after excluding the study by Li et al. [8] or Gao et al. [7]. Funnel plots for the studies comparing SMILE with FS-LASIK on the central corneal sensitivity at 1 week, 1 and 6 months were qualitatively symmetrical, indicating no obvious publication bias. The Begg’s and Egger’s test’s confirmed these results (all *p* >0.1).

## Discussion

Refractive surgeries can cause dry eye and a reduction of corneal sensation postoperatively due to a transection of the anterior corneal nerves during flap creation and laser ablation. With the help of a femtosecond laser, FS-LASIK generates a more precise and predictable corneal flap than traditional LASIK that uses microkeratome SMILE. It is a flapless surgery and better protects the corneal nerves with a lower incidence of flap-related complications.

As far as we know, this is the first meta-analysis to evaluate corneal sensitivity after FS-LASIK and SMILE. The pooled results showed that corneal sensation was significantly higher after SMILE than after FS-LASIK at 1 week, 1 and 3 months post-operation. At 6 months post-operation, although corneal sensation was numerically higher in the SMILE group, statistical significance was not achieved. Significant heterogeneity among studies was observed at 1 week, 1 and 3 months post-operation. Sensitivity analysis did not alter the results of the primary analysis, which indicated that the combined results were robust and reliable.

Many trials have found that 3–6 months are needed for corneal sensation to recover to preoperative levels after LASIK [19–22]. In this study, we confirmed that postoperative corneal sensation nearly recovered to preoperative levels during this same time frame.

The finding that SMILE exhibited a small decrease and a faster recovery of corneal sensation is biologically reasonable. First, SMILE has advantages in sustaining the integrity and smoothness of the cornea [23] Second, the outstanding superiorities of having no flap and small incisions make SMILE preserve more corneal subbasal and stroma nerves compare to surgeries with a flap. A rabbit study supports this result. More subbasal nerves longer than 200 μm were found in the SMILE group than in the LASIK group [24]. Third, nerve growth factor (NGF) in tears may influence corneal sensations after refractive surgery. NGF has been found to accelerate epithelial healing and induce migration of keratocytes [25, 26]. Another previous study demonstrated differences in NGF levels in tears between LASIK and photorefractive keratectomy-treated eyes in the early postoperative period, and the postoperative NGF concentration seemed to correlate with decreased corneal sensitivity [27]. Fourth, the concentration of proinflammatory cytokine-IL-6 in tears may also be a factor [28]. IL-6 is known to be involved in promoting corneal wound healing, and it is correlated with slower recovery [29, 30]. Furthermore, Lee et al. [31] found a significant correlation between keratocyte density after LASIK and PRK, thus an attenuated loss of keratocytes after SMILE compared with FS-LASIK might also be a reason for the fast recovery of corneal sensation.

There were several strengths of the current meta-analysis. First, we conducted a meticulous search for published studies. The study selection and data extraction were done precisely. Second, the quality assessment was conducted according to the Quality Index and all included studies had good quality. Third, the random effects model was used to obtain a relatively conservative result. Finally, tests of potential publication bias barely indicated the possibility of publication biases. Despite these advantages, some limitations to this study also exist. First, the number of included clinical trials was relatively small. Second, the included studies were almost all non-randomized trials, which increased the risk of diverse bias and decreased of reliability. Third, only one outcome (corneal sensitivity) was summarized in this study. Because adequate data was unavailable, other important outcomes could not be reviewed in our meta-analysis, such as ocular surface disease index (OSDI) and corneal biomechanics. Finally, most studies were performed in a single centre in China, hence, the results may not be able to be applied to other ethnicities. Pragmatic randomized controlled trials lasting longer and with a broader population are needed.

## Conclusion

In conclusion, the present meta-analysis suggested that central corneal sensitivity was higher after SMILE than FS-LASIK within the first three postoperative months. The differences in corneal sensitivity after SMILE and FS-LASIK were negligible 6 months after surgery.

## Abbreviations

SMILE: Small incision lenticule extraction
FS-LASIK: Femtosecond laser-assisted laser in situ keratomileusis
FS: Femtosecond laser
WMD: Weighted mean difference
PRISM: Preferred reporting items for systematic reviews and meta-analyses
ITT: Intention-to-treat

## References

[1] Stern D, Schoenlein RW, Puliafito CA, Dobi ET, Birngruber R, Fujimoto JG (1989). Corneal ablation by nanosecond, picosecond, and femtosecond lasers at 532 and 625 nm. Arch Ophthalmol.

[2] Slade SG (2007). The use of the femtosecond laser in the customization of corneal flaps in laser in situ keratomileusis. CurrOpinOphthalmol.

[3] Ambrosio RJ, Tervo T, Wilson SE (2008). LASIK-associated dry eye and neurotrophicepitheliopathy: pathophysiology and strategies for prevention and treatment. J Refract Surg.

[4] Friedlaender MH (2006). LASIK surgery using the IntraLase femtosecond laser. IntOphthalmolClin.

[5] Soong HK, Malta JB (2009). Femtosecond lasers in ophthalmology. Am J Ophthalmol.

[6] Demirok A, Ozgurhan EB, Agca A, Kara N, Bozkurt E, Cankaya KI, Yilmaz OF (2013). Corneal sensation after corneal refractive surgery with small incision lenticule extraction. Optom Vis Sci.

[7] Gao S, Li S, Liu L, Wang Y, Ding H, Li L, Zhong X (2014). Early changes in ocular surface and tear inflammatory mediators after small-incision lenticule extraction and femtosecond laser-assisted laser in situ keratomileusis. PLoS One.

[8] Li M, Niu L, Qin B, Zhou Z, Ni K, Le Q, Xiang J, Wei A, Ma W, Zhou X (2013). Confocal comparison of corneal reinnervation after small incision lenticule extraction (SMILE) and femtosecond laser in situ keratomileusis (FS-LASIK). PLoS One.

[9] Li M, Zhao J, Shen Y, Li T, He L, Xu H, Yu Y, Zhou X (2013). Comparison of dry eye and corneal sensitivity between small incision lenticule extraction and femtosecond LASIK for myopia. PLoS One.

[10] Wei S, Wang Y (2013). Comparison of corneal sensitivity between FS-LASIK and femtosecond lenticule extraction (ReLEx flex) or small-incision lenticule extraction (ReLEx smile) for myopic eyes. Graefes Arch ClinExpOphthalmol.

[11] Jiang MS, Yuan Y, Gu ZX, Zhuang SL. Corneal confocal microscopy for assessment of diabetic peripheral neuropathy: a meta-analysis. Br J Ophthalmol. 2015. 10.1136/bjophthalmol-2014-306038 25677672

[12] Moher D, Liberati A, Tetzlaff J, Altman DG (2009). Preferred reporting items for systematic reviews and meta-analyses: the PRISMA statement. J ClinEpidemiol.

[13] Higgins JP, Green S (2011). Cochrane handbook for systematic reviews of interventions. Version 5.1.0 [updated March, 2011].

[14] Wang W, Zhou M, Huang W, Zhang X (2013). Ex-PRESS implantation versus trabeculectomy in uncontrolled glaucoma: a meta-analysis. PLoS One.

[15] Cheng JW, Cai JP, Wei RL (2009). Meta-analysis of medical intervention for normal tension glaucoma. Ophthalmology.

[16] Higgins JP, Thompson SG, Deeks JJ, Altman DG (2003). Measuring inconsistency in meta-analyses. BMJ.

[17] Egger M, Davey SG, Schneider M, Minder C (1997). Bias in meta-analysis detected by a simple, graphical test. BMJ.

[18] Begg CB, Mazumdar M (1994). Operating characteristics of a rank correlation test for publication bias. Biometrics.

[19] Toda I, Asano-Kato N, Komai-Hori Y, Tsubota K (2001). Dry eye after laser in situ keratomileusis. Am J Ophthalmol.

[20] Battat L, Macri A, Dursun D, Pflugfelder SC (2001). Effects of laser in situ keratomileusis on tear production, clearance, and the ocular surface. Ophthalmology.

[21] Nassaralla BA, McLeod SD, Nassaralla JJ (2003). Effect of myopic LASIK on human corneal sensitivity. Ophthalmology.

[22] Kumano Y, Matsui H, Zushi I, Mawatari A, Matsui T, Nishida T, Miyazaki M (2003). Recovery of corneal sensation after myopic correction by laser in situ keratomileusis with a nasal or superior hinge. J Cataract Refract Surg.

[23] Sekundo W, Kunert KS, Blum M (2011). Small incision corneal refractive surgery using the small incision lenticule extraction (SMILE) procedure for the correction of myopia and myopic astigmatism: results of a 6 month prospective study. Br J Ophthalmol.

[24] Zhang F, Deng S, Guo N, Wang M, Sun X (2012). Confocal comparison of corneal nerve regeneration and keratocyte reaction between FS-LASIK, OUP-SBK, and conventional LASIK. Invest Ophthalmol Vis Sci.

[25] Micera A, Lambiase A, Puxeddu I, Aloe L, Stampachiacchiere B, Levi-Schaffer F, Bonini S, Bonini S (2006). Nerve growth factor effect on human primary fibroblastic-keratocytes: possible mechanism during corneal healing. Exp Eye Res.

[26] Sornelli F, Lambiase A, Mantelli F, Aloe L (2010). NGF and NGF-receptor expression of cultured immortalized human corneal endothelial cells. Mol Vis.

[27] Nejima R, Miyata K, Tanabe T, Okamoto F, Hiraoka T, Kiuchi T, Oshika T (2005). Corneal barrier function, tear film stability, and corneal sensation after photorefractive keratectomy and laser in situ keratomileusis. Am J Ophthalmol.

[28] Nakamura Y, Sotozono C, Kinoshita S (1998). Inflammatory cytokines in normal human tears. Curr Eye Res.

[29] Massingale ML, Li X, Vallabhajosyula M, Chen D, Wei Y, Asbell PA (2009). Analysis of inflammatory cytokines in the tears of dry eye patients. Cornea.

[30] Ebihara N, Matsuda A, Nakamura S, Matsuda H, Murakami A (2011). Role of the IL-6 classic- and trans-signaling pathways in corneal sterile inflammation and wound healing. Invest Ophthalmol Vis Sci.

[31] Lee SJ, Kim JK, Seo KY, Kim EK, Lee HK (2006). Comparison of corneal nerve regeneration and sensitivity between LASIK and laser epithelialkeratomileusis (LASEK). Am J Ophthalmol.

